# Supporting evidence for a human reservoir of invasive non-Typhoidal *Salmonella* from household samples in Burkina Faso

**DOI:** 10.1371/journal.pntd.0007782

**Published:** 2019-10-14

**Authors:** Annelies S. Post, Seydou Nakanabo Diallo, Issa Guiraud, Palpouguini Lompo, Marc Christian Tahita, Jessica Maltha, Sandra Van Puyvelde, Wesley Mattheus, Benedikt Ley, Kamala Thriemer, Eli Rouamba, Karim Derra, Stijn Deborggraeve, Halidou Tinto, Jan Jacobs

**Affiliations:** 1 Department of Clinical Sciences, Institute of Tropical Medicine (ITM), Antwerp, Belgium; 2 Nijmegen Institute of International Health, Radboud university medical centre, Nijmegen, the Netherlands; 3 IRSS/Clinical Research Unit of Nanoro (CRUN), Nanoro, Burkina Faso; 4 Centre Muraz, Bobo-Dioulasso, Burkina Faso; 5 Center for Molecular and Vascular Biology, University of Leuven (KU Leuven), Leuven, Belgium; 6 Department of Biomedical Sciences, Institute of Tropical Medicine (ITM), Antwerp, Belgium; 7 Wellcome Sanger Institute, Wellcome Genome Campus, Hinxton, Cambridge, United Kingdom; 8 Belgian National Centre for *Salmonella*, Sciensano, Brussels, Belgium; 9 Department for Global Health, Menzies School of Health Research, Darwin, Australia; 10 Université supérieur des sciences de la santé, Université polytechnique de Bobo-Dioulasso, Burkina Faso; 11 Department of Microbiology and Immunology, University of Leuven (KU Leuven), Leuven, Belgium; Institute for Disease Modeling, UNITED STATES

## Abstract

**Background:**

*Salmonella* Typhimurium and Enteritidis are major causes of bloodstream infection in children in sub-Saharan Africa. This study assessed evidence for their zoonotic versus human reservoir.

**Methods:**

Index patients were children with blood culture confirmed *Salmonella* infection recruited during a microbiological surveillance study in Nanoro, rural Burkina between May 2013 and August 2014. After consent, their households were visited. Stool from household members and livestock (pooled samples per species) as well as drinking water were cultured for *Salmonella*. Isolates with identical serotype obtained from index patient and any household sample were defined as “paired isolates” and assessed for genetic relatedness by multilocus variable number tandem-repeat analysis (MLVA) and whole-genome sequencing (WGS).

**Results:**

Twenty-nine households were visited for 32/42 (76.2%) eligible index patients: two households comprised two index patients each, and in a third household the index patient had a recurrent infection. Among the 32 index patients, serotypes were *Salmonella* Typhimurium (n = 26), *Salmonella* Enteritidis (n = 5) and *Salmonella* Freetown (n = 1). All Typhimurium isolates were sequence type (ST)313. Median delay between blood culture sampling and household visits was 13 days (range 6–26). *Salmonella* was obtained from 16/186 (8.6%) livestock samples (13 serotypes) and 18/290 (6.2%) household members (9 serotypes). None of the water samples yielded *Salmonella*. Paired *Salmonella* Typhimurium isolates were obtained from three households representing four index patients. MLVA types were identical in two pairs and similar in the third (consisting of two index patients and one household member). WGS showed a strong genetic relatedness with 0 to 2 core genome SNPs difference between pairs on a household level. Livestock samples did not yield any *Salmonella* Typhimurium or *Salmonella* Enteritidis, and the latter was exclusively obtained from blood culture. Other serotypes shared by human and/or livestock carriers in the same household were *Salmonella* Derby, Drac, Tennessee and Muenster.

**Conclusions/Significance:**

The current study provides further evidence of a human reservoir for invasive non-Typhoidal Salmonella (iNTS) in sub-Saharan Africa.

## Author summary

Non-Typhoidal *Salmonella*—particularly *Salmonella* Typhimurium sequence type (ST) 313—are among the most common causes of bloodstream infection in children in sub-Saharan Africa. To assess transmission and reservoir of this invasive serotype we performed a blood culture surveillance study among children < 15 years (index patients) in Nanoro, rural Burkina Faso. In case of blood culture confirmed invasive Non-Typhoidal *Salmonella* (iNTS) infection among index patients a household survey was performed, during which stool samples from household members, stool samples from livestock and water samples were obtained and cultured for *Salmonella*. *Salmonella* isolates obtained from blood culture of index patients were compared for genetic relatedness with *Salmonella* isolates obtained from samples taken from their corresponding household. In three households we found *Salmonella* Typhimurium ST 313 among both index patient and the stool sample of a household member, we found a strong genetic relatedness within each pair. There were no matching serotypes between index patients and livestock samples or water samples within households. Clusters from other serotypes were restricted to stool samples from household members and different species of livestock. Our results support the theory that iNTS has a human reservoir.

## Introduction

Non-Typhoidal *Salmonella* (NTS) serotypes are among the most common causes of bacterial bloodstream infections in sub-Saharan Africa [1–10] with an estimated 680.000 deaths per year, mainly among children below the age of five [1]. The high prevalence of invasive Non-Typhoidal *Salmonella* (iNTS) disease in sub-Saharan Africa is in sharp contrast to industrialized countries, where NTS serotypes mainly cause self-limiting gastroenteritis [11] occasionally progressing to invasive infection. Throughout the manuscript the abbreviation “NTS” is used to refer to the group of pathogens while the abbreviation “iNTS” refers to the invasive human disease. The two serotypes which are most commonly implicated in iNTS in Sub-Saharan Africa are *Salmonella enterica* subspecies *enterica* Typhimurium (in particular Sequence Type (ST) 313 lineage II [12]) and *Salmonella enterica* subspecies *enterica* Enteritidis (hereafter referred to as *Salmonella* Typhimurium and *Salmonella* Enteritidis). Important risk factors are young age, *Plasmodium falciparum* malaria, malnutrition and HIV-infection [13, 14].

While NTS gastroenteritis is generally considered a zoonotic disease with a broad host range transmitted through consumption of contaminated meat and dairy products, the reservoir, source of transmission and mode of transmission of iNTS in sub-Saharan Africa remain unknown [15]. Recent studies found evidence that the pathogens involved in iNTS in sub-Saharan Africa have genetically adjusted to become more invasive and human adapted [15], suggesting a potential human reservoir. This was also suggested by a study performed in semi-urban Kenya in 2006 which found that isolates from iNTS patients were similar in antibiotic resistance pattern and pulsed-field gel electrophoresis (PFGE) profile to isolates of the same serotype obtained from stool samples of household members, while isolates from environmental and livestock stool sampling were distinctly different [7]. The arrival of new molecular technologies with higher resolution and discriminatory power such as Multilocus variable number tandem-repeat (MLVA) analysis and whole genome sequencing (WGS) to assess genetic relatedness between isolates has the potential to provide additional evidence to the reservoir of the iNTS reservoir. We therefore performed a household-based study to assess the reservoir of iNTS in a rural community in Burkina Faso applying a combination of microbiological techniques with WGS and MLVA analysis.

## Methods

### Study site and population

Nanoro is a village in the province of Boulkiemdé, in the Centre-West region of Burkina Faso at 85 km of its capital Ouagadougou. This site was selected because of its rural nature in combination with its high prevalence of iNTS among children [16–18]. The study was conducted at the Clinical Research Unit of Nanoro (CRUN), situated on the compound of the district hospital “Centre Médical avec Antenne Chirurgicale” (CMA) Saint Camille. The Nanoro Health and Demographic Surveillance System (HDSS) catchment area in which CRUN operates covers a surface area of 594.3 km^2^ with a population density of 125/km^2^. In Burkina Faso, like in large parts of sub-Saharan Africa, the majority of the rural population live in close proximity to their livestock [19] mostly consisting of goat, sheep, poultry and donkeys. About 90% of the population is engaged in subsistence agriculture [20]. Families traditionally live together in compounds housing 5 to 50 family members and their livestock. Improved sanitary facilities as defined by WHO criteria [21] are available in 15% of households [20, 22]. Most households have unimproved water wells close to their compound and use water buckets or 40-liter containers, which are replenished daily for daily use. Improved water sources are available within approximately 1 km from each compound, however maintenance is irregular. Refrigerators are rare: staple foods, fruits, vegetables and meat products are generally cooked and consumed the day they are bought. The area has a tropical climate with a rainy season lasting from June to September, *Plasmodium falciparum* malaria is hyperendemic with a seasonal pattern.

### Study design

We conducted a surveillance study among febrile patients between 2 months and 15 years old to assess the population-based incidence of iNTS in this area, details on the primary outcomes have been published elsewhere [16]. For the current study, all households of patients with blood culture confirmed iNTS infection living in the HDSS area were eligible for inclusion (referred to as “index patients”). Households of index patients were visited after consent and drinking water samples, stool samples from household members and livestock were collected and cultured for *Salmonella*. *Salmonella* isolates with identical serotype obtained from the index patient and any other source within their corresponding household were defined as “paired isolates” and, in case of Typhimurium or Enteritidis serotypes, assessed for genetic relatedness through multilocus variable number tandem-repeat analysis (MLVA) and whole genome sequencing (WGS). “Clusters” of *Salmonella* isolates were defined as two or more isolates of the same serotype obtained from multiple sources within one household, but excluding the previously defined paired isolates. They comprised multiple index patients, household members and animals, multiple animals or multiple household members. Recurrent infection was defined as a second episode with a *Salmonella* isolate obtained from blood culture at least 14 days after the first was identified.

### Index patients with *Salmonella* isolates obtained from blood cultures

As part of a surveillance study, blood cultures were sampled in children presenting to the pediatric ward of CMA hospital and one Healthcare Centre in the HDSS catchment area, between May 2013 and August 2014 (for indications, sampling and laboratory work-up of the blood cultures see reference [16]). Basic demographic and clinical data, including age, sex, residence, symptoms and prior treatment, were recorded. According to local Burkinabé policy [19], HIV testing was only done on clinical indication. Patients with blood culture confirmed iNTS were selected for inclusion.

### Household visits

Households of index patients whose parent/ legal guardians consented were visited. Household visits entailed the sampling of (i) stool samples from all consenting household members and visitors who spent at least one night in the same house with the index patient between 72 and 24 hours before onset of symptoms in the index case (ii) pooled stool samples from each species of livestock living inside or around the compound as explained below (iii) water samples from the households main water source used for drinking and food preparation. Prepared foods were not sampled since it is culturally not acceptable to use food for other purposes than consumption.

### Stool sampling of household members and livestock

The number of household members and visitors as well as the numbers of livestock per species in the compound was recorded. History of fever, diarrhea or antimicrobial use was not recorded. Household members were instructed to collect a stool sample in a sterile collection cup with lid. A single sample of a minimum of 10 grams was required.

Pooled stool samples were collected from each livestock species. Pooled samples consisted of 25 grams of stool obtained from a maximum of 5 animals from the same species [23]. Acceptable samples included freshly deposited stools (observed) or rectal fecal samples collected by a veterinarian. One sample was collected if the compound had 5 or less animals of the same species, two samples for 6–10 animals, 3–4 samples for 11–30 animals, 5 samples for 31–100 animals and 6 samples for more than 100 animals.

After collection, all human and animal samples were stored in a coolbox and transported to the laboratory within 8 hours after collection.

### Collection of water samples

The main sources of drinking water were identified by one of the household members. Water was sampled directly from the main source (e.g. borehole, wells, sachets, barrages), stored water was not sampled. In case of clear water 1000 ml was collected in sterilized flasks with 6 ml 1% Na_2_S_2_O_3_ solution. Turbid water from open water reservoirs was sampled using a Moore swab—a swab attached to a 10 m string [24]. The swab was submerged in the water source for 48 hours after which it was collected and placed in a sterile bag. All samples were placed in a cool box, transported to the laboratory and processed within 6 hours of collection.

### Laboratory work-up of stool and water samples

Stool samples were diluted in a ratio of 1:10 and subsequently incubated on Buffered Peptone Water (Oxoid, Basingstoke, UK) and Selenite Broth (SB). After overnight incubation, 10μl of Selenite Broth was inoculated onto two Salmonella-Shigella (SS) agar plates and incubated overnight. Clear water samples were first filtered using the membrane filtration technique with filter pore size 0.45μm (Sartorius, Goettingen, Germany). Both filters and Moore swabs were incubated overnight in Selenite Broth, after which a 10μl volume was subcultured onto two SS agar plates. All agar plates were incubated for 12–18 hours at 35–37°C.

Lactose negative and H_2_S producing colonies growing on SS agar were considered suspected for *Salmonella* and processed: at least two of each morphologically similar colonies per agar plate were subcultured on a biochemical gallery including Kligler Iron Agar (Oxoid) Motility-Lysine-Indole agar (Becton Dickinson), Simmons Citrate agar (Oxoid) and urease and ornithine reactions (DiaTabs, Rosco, Taastrup, Denmark). All biochemically suspected *Salmonella* isolates were serotyped using antisera (Prolab Diagnostics, Richmond Hill, Canada). Isolates were stored on Microbank cryogenic vials (Prolab Diagnostics) at -80°C. Subcultures of all isolates were shipped to the Institute of Tropical Medicine of Antwerp (ITM) on Trypticase Soy Agar (TSA) (Oxoid) and the Belgian reference center for *Salmonella* (Sciensano, Brussels) for serotype confirmation, MLVA typing and WGS analysis.

### Multilocus variable number tandem-repeat (MLVA) tying

Paired isolates obtained from index patients and households were sent to the Belgian National Centre for *Salmonella* (Sciensano, Brussels, Belgium) for multi-locus variable-numbers tandem repeat analysis (MLVA) as previously described [25–27]. MLVA profiles were attributed based on the number of tandem repeats on five loci (STTR9-, STTR5-, STTR6-, STTR10-, STTR3). Identical MLVA types of the Typhimurium serotype were defined as isolates with variation in none or one of the rapidly changing loci (STTR5, STTR6 and STTR10) and no variation in the stable loci (STTR3 or STTR9) [28].

### Whole genome sequencing (WGS) analysis

All *Salmonella* Typhimurium isolates from this study were subjected to whole genome sequencing (**Supplementary Data 1**). DNA was extracted using the Gentra Puregene kit (Qiagen, Germany), subjected to WGS on the Illumina 1500 HiSeq platform at the UA sequencing facility. Genomics MLST was determined using the SRST2 software. Genomic context was provided by including five genome sequences from *Salmonella* Typhimurium ST313 lineage II, originating from The Democratic Republic of the Congo (n = 1), Mali (n = 2), Malawi (n = 1) and Nigeria (n = 1) [29, 30]. All sequences were mapped on the *Salmonella* Typhimurium D23580 reference sequence (Accession Number = NC_016854), using SMALT v0.7.4. Variation detection was performed using samtools mpileup v0.1.19 with parameters “-d 1000 -DSugBf” and bcftools v0.1.19 [31]. A pseudo-genome was constructed by substituting the base call at each site (variant and non-variant) in the BCF file into the reference genome and any site called as uncertain was substituted with an N. Insertions with respect to the reference genome were ignored and deletions with respect to the reference genome were filled with N’s in the pseudo-genome to keep it aligned and the same length as the reference genome used for read mapping. Plasmids and recombinant regions (prophages and CRISPR sequences) in the chromosome were removed from the alignment. SNP sites were extracted from the alignment using snp-sites [32] and used to construct a maximum likelihood phylogeny. RAxML v8.2.8 [33] with substitution model GTRCAT. Support for nodes on the trees was assessed using 100 bootstrap replicates. The number of SNPs on the branches were calculated using Sankoff Parsimony ([34–36]. The tree was rooted on *Salmonella* Typhimurium DT2B [29]. Trees were visualized using iTOL [37]. The sequence reads are available at the Sequence Read Archive (SRA) with accession number PRJNA544902.

### Data collection and statistical analysis

Clinical data from the index patients were entered into an Epi-InfoTM^7^ database (CDC, Atlanta, Georgia, USA) by double data entry. Household visits done more than 30 days after enrolment of the index case were excluded from analysis. Data from the household visits were recorded in an Excel database (Microsoft, Richmond, US). After conformity check, statistical analysis was done using Stata 12 (Stata Corp., College Station, TX, USA). Differences in proportions were compared using the Chi-square test, chi-square test for trends or the two-tailed Fisher’s exact test. Continuous data were compared using a students’ t-test or Wilcoxon rank test in case of not-normally distributed data. A p-value of 0.05 was considered as statistically significant.

### Ethics statement

The study was conducted according to the principles expressed in the Declaration of Helsinki [38] and was approved by the national ethics committee of Burkina Faso (Comité d’Ethique pour la Recherche en Santé (CERS), deliberation N°2013-01-08 from January 09^th^ 2013), the institutional review board of the ITM, Antwerp (Reference 843/12 from December 4^th^, 2012) and the ethics committee of the University Hospital of Antwerp (January 01^st^, 2013). Written informed consent for patient participation and blood culture was obtained from parents or legal guardians prior to inclusion. Separate informed consent was obtained from the house elder for the sampling of water and animal stools during the household surveys. Household members participating in the study were asked for oral informed consent. In case of illiteracy an independent witness was present.

## Results

### Index patients

Between May 2013 and August 2014 a total of 1,339 blood cultures were sampled from patients below the age of 5. A complete overview of blood culture pathogens isolated in the course of the surveillance study has been published elsewhere [16]. In total 42 patients with iNTS met inclusion criteria to the current study. Serotypes included *Salmonella* Typhimurium (n = 33, 78.6%), *Salmonella* Enteritidis (n = 7, 16.7%), *Salmonella* Brancaster (n = 1) and *Salmonella* Freetown (n = 1). An overview of demographic data, serotype distribution and co-infections of the index patients is presented in Table 1.

**Table 1:** Overview of Index patients obtained from the HDSS catchment area during the surveillance period.

	n = 42
**Demographic data**	
Age in months (median (IQR))	21 (12–41)
Age in years (median (IQR))	1.8 (1.0–3.4)
Male (n (%))	23 (52.8)
Temperature (median (IQR))	39.2 (38.0–39.6)
Pulse (median (IQR))	131 (107–149)
Systolic BP (median (IQR))	96.5 (85–110)
Diastolic BP (median (IQR))	57.5 (49.0–72.0)
**Co-infections**	
Malaria positive (n (%))	18 (42.8)
Malaria–density/μL (median (IQR))	6479 (2248–40775)
HIV (n (%))	2 (4.8)
**Serotype distribution**	
*Salmonella* Typhimurium (n (%))	33 (78.6)
*Salmonella* Enteritidis (n (%))	7 (16.7)
Other iNTS serotypes (n (%))	2 (4.8)

### Household visits

Two households (household 5 and 28) comprised of two index patients each, presented with a two- and four-day difference respectively. In a third household (household 3), the index patient had a recurrent infection three weeks after the initial infection. For these households only one visit per household was performed. For eight patients no household visit was performed because household members refused to participate (n = 2) or for logistic reasons (n = 6). Additionally, data from two households were excluded from the analysis because the visits were made more than 30 days after the index patient had been enrolled. The current analysis therefore included 29 households representing 32 out of 42 (76.2%) eligible index patients.

**Fig 1:**
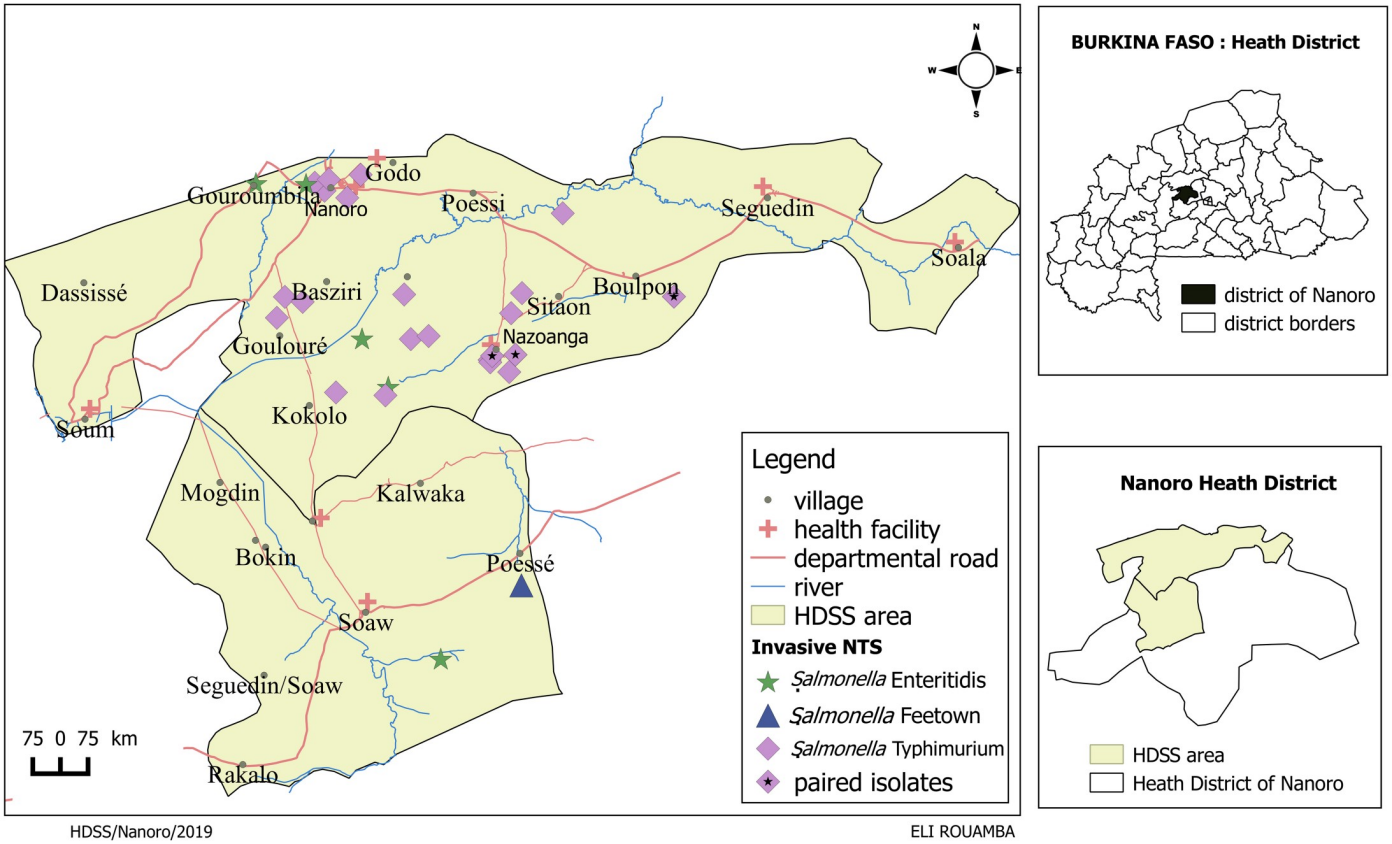
Geographic distribution of *Salmonella* isolates obtained from blood culture and household visits performed within the HDSS catchment area.

The 32 index patients comprised the following serotypes: *Salmonella* Typhimurium (n = 26, 81.3%), *Salmonella* Enteritidis (n = 5, 15.6%) and *Salmonella* Freetown (n = 1, 3.1%). Fig 1 and Table 2 show the geographical distribution of isolated *Salmonella* serotypes and sampled households. The index patients included in the analysis were representative for the total number of blood culture confirmed iNTS patients obtained from the HDSS in the surveillance study (Table 3). The median age of included index patients was 1.7 (range 0.7–5.0) years, compared to 1.8 (range 0.2–4.5) years among non-included index patients (p = 0.83). The index patients and serotypes included in the analysis were representative for the total number of blood culture confirmed iNTS patients.

**Table 2:** Proportion and serotype distribution of non-Typhoidal *Salmonella* isolates recovered from blood of index patients living in the HDSS area and stool samples from their corresponding household (household members and livestock).

May 2013-August 2014	Serotype of index patients for whom household visit was performed	Serotypes from stool samples obtained during household visits **	
		Household members	Livestock
**Total numbers of samples**		n = 290	n = 186
**Numbers of NTS isolates (proportion of carriage %)**	32 patients in 29 households*	18 (6.2)	16 (8.6)
**Numbers per serotype**	n (%)	n (%)	n (%)
*Salmonella* Typhimurium	26 (81.3)	3 (16.7)	-
*Salmonella* Enteritidis	5 (15.6)	-	-
*Salmonella* Freetown	1 (3.1)	-	-
*Salmonella* Bredeney	-	1 (5.6)	-
*Salmonella* Carmel	-	1 (5.6)	-
*Salmonella* Drac	-	2 (11.1)	-
*Salmonella* Derby	-	4 (22.2)	3 (18.8)
*Salmonella* Ebrie	-	1 (5.6)	-
*Salmonella* Korlebu	-	1 (5.6)	-
*Salmonella* Muenster	-	1 (5.6)	1 (6.3)
*Salmonella* Tennessee	-	1 (5.6)	1 (6.3)
*Salmonella* I 41:z10:-	-	3 (16.7)	-
*Salmonella* Binningen	-	-	1 (6.3)
*Salmonella* Brancaster	-	-	1 (6.3)
*Salmonella* Give	-	-	1 (6.3)
*Salmonella* Llandorff	-	-	1 (6.3)
*Salmonella* Poona	-	-	2 (12.5)
*Salmonella* Schwarzengrund	-	-	1 (6.3)
*Salmonella* Vilvorde	-	-	1 (6.3)
*Salmonella* non-typeable	-	-	1 (6.3)
*Salmonella* I 3,19:z:-	-	-	1 (6.3)
*Salmonella* I 4:b:-	-	-	1 (6.3)

NTS: non-Typhoidal *Salmonella* | HDSS: Health and Demographic Surveillance System

* In two households 2 index patients were siblings infected with *Salmonella* Typhimurium living in the same household and in a third household, the index patient had a recurrent infection three weeks after the initial infection

** Household members: one sample per household member| livestock: pooled samples

**Table 3:** Characteristics of included versus excluded index patients.

	Included index patients (n = 32)	Excluded index patients (n = 10)	p-value
**Age in years (range)**	1.7 (0.7–5.0)	1.8 (0.2–4.5)	.83
**Male (%)**	19 (59.9)	4 (40.0)	.28
**Female (%)**	13 (40.1)	6 (60.0)	.28
**Serotype distribution**			
Salmonella Typhimurium (%)	26 (81.3)	7 (70.0)	.45
Salmonella Enteritidis (%)	5 (15.6)	2 (20.0)	.75
Other serotypes (%)	1 (3.1)	1 (10.0)	.37
Month of sampling*			
December-June (%)	21 (65.6)	8 (80.0)	.39
July-November (%)	11 (34.4)	2 (20.0)	.39

Data represent numbers unless otherwise stated.

* Rainy season lasts from July-September, dry season lasts from October-June.

The median number of days between blood culture sampling of the index case and the corresponding household visit was 13 days (range 6–26). In total 21/29 (72.4%) visits were done during the rainy season and the start of the dry season (July-November). The remaining visits were distributed evenly between December and April.

Households consisted of a median of 9 household members (range 3–43). The median age of participating household members was 15 years (range 0–91 years) and 41.3% of them were male. Out of 336 household members, 290 (86.3%) provided a stool sample. Reasons for non-participation were individual refusal (n = 13), absence from home at the days of sampling (n = 25), unable to produce (n = 2) and non-specified reasons (n = 6). In one household all household members refused to provide a stool sample, the household was not excluded because the family did consent to sample livestock stool and drinking water. Additionally, one household did not have any livestock.

Households had a median of four different species of animals (range 2–6) and a median of five (range 2–17) pooled samples were taken per household. A total of 186 pooled stool samples from livestock were collected among 28/29 households: 10 from cows, 39 from sheep, 32 from goat, 23 from donkeys, 27 from pigs and 55 from poultry.

A total of 30 water samples were collected among 25 households: 18 boreholes, 8 wells, 2 water reservoirs and 2 water bags. In three households the local water source was out of function.

### Serotype distribution among household members and livestock

None of the water samples yielded *Salmonella* isolates. Tables 2 and 4 provide an overview of the proportions and serotype distribution of NTS serotypes among index patients and human or livestock carriers obtained from households. No mixed infections with multiple serotypes in one sample were observed. Overall, the proportion of stool samples with growth of *Salmonella* among household members and livestock was 6.2% (n = 18) and 8.6% (n = 16) respectively; proportions ranged from 6.3% amongst goats to 10.3% amongst sheep. Proportions of *Salmonella* carriage and serotype distribution did not differ between species (all p>0.05). In humans, proportions of *Salmonella* carriage tended to increase with age with a positivity rate of 2.4%, 5.0% and 8.1% among household members < 5 years old, 5 to 14 years old and ≥ 15 years old respectively, however this did not reach statistical significance (Chi-square for trends 2.18, p = 0.14).

**Table 4:** Overview of samples taken during household surveys, growth of NTS in stool samples and serotype distribution. Numbers represent individual household members or, in case of livestock, pooled animals from a single species.

Source	Household member age	Nrs (%) of (pooled) samples taken	Nrs (%) of samples containing NTS	NTS serotype (n = 1 isolate unless otherwise mentioned)
**Household members** (Stool)*		n = 290	n = 18	
	≤ 4 yrs	41 (14.1)	1 (2.4)	Typhimurium
	5–14 yrs	101 (34.8)	5 (4.9)	Typhimurium
				Derby (n = 2)
				Drac
				Tennessee
	≥ 15 yrs	148 (51.0)	12 (8.1)	Typhimurium
				Derby (n = 2)
				Carmel
				Drac
				Bredeney
				Muenster
				Ebrie
				Korlebu
				I4:z10:- (n = 2)
				I 4:b:-
**Livestock** (Stool)*	Species	**n = 186**	**n = 16**	
	Cow	10 (5.3)	1 (10.0)	Schwarzengrund
	Sheep	39 (21.0)	4 (10.3)	Derby
				Poona (n = 2)
				Tennessee
	Goat	32 (17.2)	2 (6.3)	Derby
				Non-typeable
	Donkey	23 (12.4)	2 (8.7)	Derby
				Vilvorde
	Pig	27 (14.5)	2 (7.4)	Give
				Llandorff
	Poultry	55 (29.6)	5 (9.1)	Binningen
				Brancaster
				Muenster
				I 3,19:z:-
				I 4:b:-
**Drinking water**	Water source	**n = 30**	**n = 0**	
	Boreholes	18 (60.0)	-	NA
	Wells	8 (26.6)	-	NA
	Reservoirs	2 (6.7)	-	NA
	Sachet	2 (6.7)	-	NA

NTS: non-Typhoidal Salmonella | Nrs: Numbers | yrs: years of age

* Household members: one sample per household member| livestock: pooled samples

There was a large variety of *Salmonella* serotypes among both stool samples from humans and livestock (9 and 11 serotypes respectively). *Salmonella* Enteritidis was not found in any of the stool cultures and *Salmonella* Typhimurium (n = 3) was recovered exclusively from human samples (Table 2). Three serotypes (Tennessee, Muenster and Derby) were obtained from both human samples and livestock, with serotypes Derby and Tennessee occurring in ungulates and serotype Muenster in poultry (Table 4).

### Paired *Salmonella* isolates from index patients and household members

**Fig 2:**
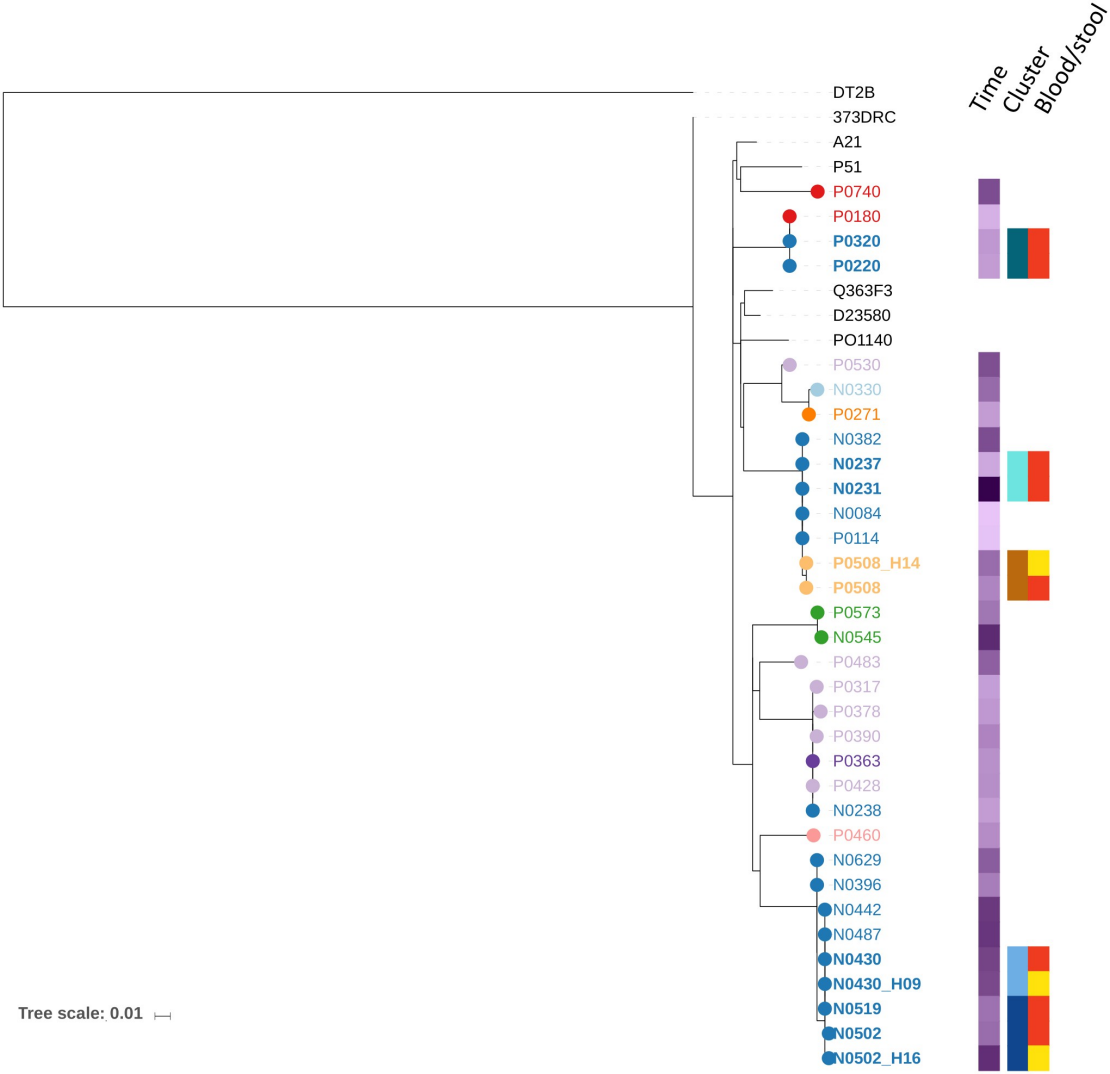
Phylogenetic tree of *Salmonella* Typhimurium isolates obtained from blood culture and household visits performed within the HDSS catchment area. The phylogenetic tree is based on mapping *Salmonella* Typhimurium ST313 lineage II to reference strain D23580. The sequencing data from this study is analyzed in the context of 5 diverse African ST313 lineage II strains included as references, isolate DT2B was included to root the phylogenetic tree [30]. The branch tips and names from the isolates obtained in this study are color-coded based on village of collection. Time: The month of isolation is shown as a color gradient in purple. Cluster: clusters and pairs are color coded on household level. Blood/Stool: origin of the paired and clustered isolates whereby red represents blood and yellow represents stool.

Paired isolates occurred exclusively for *Salmonella* Typhimurium; three pairs were found including four index patients and three household members living in three households (households 17, 24 and 28). The four index patients were less than 4 years of age and presented with fever and abdominal complaints (Table 5). One patient had a coinciding microscopic malaria infection. The corresponding human carriers were a 44-year-old male, a 10-year-old female and a 13-month-old male. MVLA typing of the seven *Salmonella* Typhimurium isolates revealed four MLVA types. In two households (households 17 and 24), the MVLA types of the index patients were identical to the MLVA type of the corresponding household member (Table 5). In household 28, the two index patients and corresponding household member had MLVA types which differed in two out of three rapidly changing loci. Paired isolates from index patients and household members in the 3 households (17, 24, 28) showed respectively 0, 0 and 2 SNPs difference in their core genomes (S1 Fig). The MLVA type of the bloodstream isolate of one of the siblings from household 28 was identical to the MLVA obtained from household 24. The phylogenetic tree shows a close genetic relationship between these isolates and two additional isolates obtained from blood culture in different households (Fig 2).

**Table 5:** Paired *Salmonella* isolates obtained from index patients and corresponding household members.

Household visit	Days between sampling*	Index patient				Household member		
		Age (years)	Sex	Malaria diagnosis	MLVA type	Age (years)	Sex	MLVA type
**17**	10	3.8	M	negative	2-8-7-9-0210	44	M	2-8-7-9-0210
**24**	8	1.1	M	positive	2-7-10-8-0210	10	F	2-7-10-8-0210
28**	11	3.5	F	negative	2-7-12-NA-0210	1	M	2-7-17-8-0210
	7	0	M	negative	2-7-10-8-0210			

MLVA: multilocus variable number tandem-repeat analysis

* Days between sampling: Number of days between inclusion of the index patient and the household visit.

** Two index patients from the same family, presenting with a 4-day delay.

These four households were geographically close (Fig 1) and all isolates were obtained within a two-month window. For the paired isolates, the genetic relationship between isolates was always strongest within the pair compared to any other analyzed isolate (Fig 2). Whole genome sequencing further showed that all *Salmonella* Typhimurium isolates from this study were part of ST313 clonal lineage II.

### Clusters of NTS isolates (others than pairs)

Apart from the siblings with *Salmonella* Typhimurium in household 28, there was a second cluster of two siblings with blood culture confirmed *Salmonella* Typhimurium (household 5): a boy of 11 months and his sister of 4.7 years presented within two days of each other, but no matching isolates were obtained from other household samples. The isolates from the index patients of household 5showed a strong genetic relatedness (Fig 2). Additionally, the isolates from the patient with recurrent infection (household 3) were also strongly related.

Other serotypes apart from *Salmonella* Typhimurium were also observed to form clusters. There were two household clusters of *Salmonella* Derby (four and two members respectively) and one household cluster (two household members) of *Salmonella* Drac. *Salmonella* Derby was found in seven household members among three households (four, two and one carrier in each household). *Salmonella* Drac was furthermore found among two out of twenty-five household members in one household. Three serotypes (*Salmonella* Brancaster, *Salmonella* Tennessee and *Salmonella* Muenster were each found among human carriers and livestock.

## Discussion

The present study provides evidence that iNTS in sub-Saharan Africa may rely on a human reservoir. *Salmonella* Typhimurium was isolated from four index patients and their respective household members, but was not obtained from any livestock living in the same household nor from any drinking water samples. At the same time several clusters of *Salmonella* serotypes other than *Salmonella* Typhimurium and *Salmonella* Enteritidis were found among stool samples of livestock and household members.

In the United States *Salmonella* Enteritidis gastroenteritis is commonly associated with consumption of raw eggs and poultry [39]. The widely recognized zoonotic nature of NTS gastroenteritis in industrialized countries prompted the assumption that iNTS in sub-Saharan Africa might also have a zoonotic reservoir and transmission. The current study found limited overlap between serotypes obtained from human and livestock sources. The relatively low number of animals sampled in our study may partially explain this result. However, our results are in line with a previous study performed in Tanzania in 2007 [40]. Dione et al assessed the genetic correlation between NTS serotypes isolated from gastroenteritis cases and livestock in Tanzania. While they found some overlap between serotypes obtained from human and livestock stool samples, just like we did, the samples showed wide genetic variation suggesting that human and livestock strains of serotypes each had their own preferred reservoir.

Other animals which were not sampled in the current study such as reptiles and bush mammals may also be considered as a reservoir for iNTS. Geckos and lizards are particularly common in the Nanoro area and have the potential to access and contaminate food or water sources. It would be interesting for future (prospective) studies to look at these potential reservoirs.

It is not excluded that a common source (e.g. food, water sample,) infected both household member well as the index patient. This notwithstanding, we demonstrated that household members of index patients excreted the same *Salmonella* Typimurium isolate as the one causing invasive infection. Further research should therefore address food and water sources as in addition to a common source of transmission, they may also be a substrate for multiplication of Salmonella up to the infectious dose. In 2006 Kariuki et al found evidence for a human reservoir of both *Salmonella* Enteritidis and *Salmonella* Typhimurium [7]. The results of our study are in line with theirs and contribute to the message by demonstrating that the paired isolates as well as clusters among index patients were genetically identical. These results support the hypothesis that multiple strains of NTS, in particular *Salmonella* Typhimurium and *Salmonella* Enteritidis, have adapted to the human host. *Salmonella* Typhi has lost a number of accessory genes which were used by the host immune system to enable a stronger immune response [41]. It has been theorized that *Salmonella* Typhimurium ST-313 and *Salmonella* Enteritidis have undergone similar adaptations [12, 15].

There were several factors which may have decreased the yield from household visits: (i) time between exposure and symptoms of the index patient (incubation time) (ii) time needed for blood culture growth and identification of *Salmonella* (iii) accessibility of the household sites (iv) time to mobilize the field outreach team and (v) single stool sampling. The incubation time of infection by NTS (established for intestinal salmonellosis) is estimated at 12 hours to 3 days [42], and the time from blood culture sampling to identification and reporting of NTS may take 4 days.

Additionally, logistical constraints in the present study not only limited the area in which household visits could be performed but also caused a delay in several household visits, leading to exclusion from analysis of some household visits. This was particularly true during the rainy season when roads may be difficult or impossible to access. Human carriage for *Salmonella* has been reported as low as 10^6^ CFU/gram stool [43] and sampling on consecutive days may significantly increase yields [44]. Sampling on multiple days was however not feasible in the social context of the current study.

Drinking water is an important source of transmission in *Salmonella* Typhi [3] and has been suggested for iNTS as well. In addition to drinking contaminated water, food washed with- or cooked in- contaminated water could also be a source of transmission. In the current study none of the water samples yielded *Salmonella*. This may in part be due to the delay to water sampling as previously mentioned, the notoriously low yield from water samples due to the inactive state *Salmonella* (Typhi) develops when outside of the human body, and the fact that most of our water samples were clear; *Salmonella* adheres to sediment in water to promote survival and replication [45]. The use of buffered peptone water as a separate enrichment step for water culture might have increased the yield from water samples.

Sampling prepared foods would have been illustrative but was considered not culturally appropriate.

Intestinal carriage of *Salmonella* Typhi has been suggested to last for several decades and is associated with colonization of the gallbladder causing intermittent fecal shedding [46]. Carriage rates of NTS among household members in the current study was 6.2%. This is in line with previous literature where asymptomatic carriage rates of 2.4% [47] 3.9% [48] and 6.9% [7] among humans are described. Interestingly, paired asymptomatic human household carriers were across an age range from very young to adult, which shows that the stool excretion of *Salmonella* Typhimurium is not always associated with overt disease and serious symptoms. Patients recovered from NTS gastroenteritis continue to shed NTS in their stool for a median of 5 weeks after infection, with 1% continuing to shed for as long as 1 year after infection [49]. There is however very limited knowledge on the carriage of NTS during and after invasive NTS infection. Future research could include longitudinal sampling of both index cases and household members to address this important subject.

### Conclusion

In conclusion, the present study shows genetic relatedness between NTS isolates obtained from patients with iNTS and stool isolates obtained from household members. These results add evidence to the hypothesis that the human intestines are the reservoir of iNTS.

## Supporting information

S1 Data List of *Salmonella* Typhimurium isolates analysed in this study. (XLSX)

S1 Fig "Phylogenetic tree of *Salmonella* Typhimurium isolates obtained from blood culture and household visits performed within the HDSS catchment area with SNP differences assigned. The phylogenetic tree is based on mapping on Salmonella Typhimurium ST313 lineage II to reference strain D23580. The sequencing data from this study is analyzed in the context of 5 diverse African ST313 lineage II strains included as references, isolate DT2B was included to root the phylogenetic tree [30]. Numbers of SNPs are annotated on the branches. For the paired and clustered isolates blood isolates are indicated with red and stool isolates with yellow".
(PDF)
