# Supplementary figures and images for: Supporting evidence for a human reservoir of invasive non-Typhoidal Salmonella from household samples in Burkina Faso
Source: PLoS Negl Trop Dis. 2019 Oct 14;13(10):e0007782. doi: 10.1371/journal.pntd.0007782 (PMC6812844; doi:10.1371/journal.pntd.0007782)

Tree scale: 10

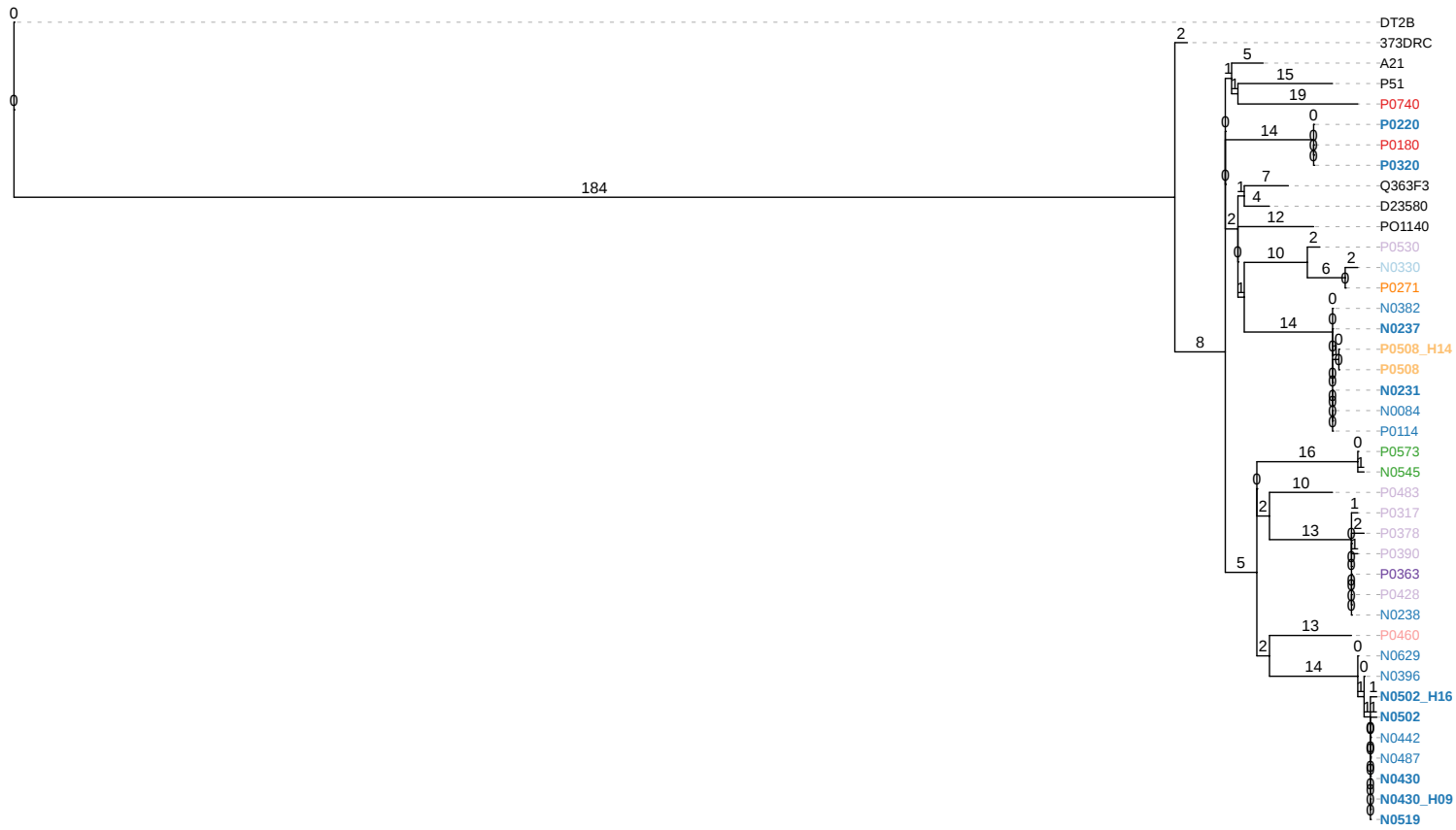

Supplement: S1 Fig — The phylogenetic tree is based on mapping on Salmonella Typhimurium ST313 lineage II to reference strain D23580. The sequencing data from this study is analyzed in the context of 5 diverse African ST313 lineage II strains included as references, isolate DT2B was included to root the phylogenetic tree [30]. Numbers of SNPs are annotated on the branches. For the paired and clustered isolates blood isolates are indicated with red and stool isolates with yellow". (PDF) [file pntd.0007782.s002.pdf]
